# Integration of Virtual Reality in the Control System of an Innovative Medical Robot for Single-Incision Laparoscopic Surgery

**DOI:** 10.3390/s23125400

**Published:** 2023-06-07

**Authors:** Florin Covaciu, Nicolae Crisan, Calin Vaida, Iulia Andras, Alexandru Pusca, Bogdan Gherman, Corina Radu, Paul Tucan, Nadim Al Hajjar, Doina Pisla

**Affiliations:** 1 Research Center for Industrial Robots Simulation and Testing—CESTER, Technical University of Cluj-Napoca, 400114 Cluj-Napoca, Romania; 2 Department of Urology, “Iuliu Hatieganu” University of Medicine and Pharmacy, 400012 Cluj-Napoca, Romania; 3 Department of Internal Medicine, “Iuliu Hatieganu” University of Medicine and Pharmacy, 400012 Cluj-Napoca, Romania; 4 Department of Surgery, “Iuliu Hatieganu” University of Medicine and Pharmacy, 400012 Cluj-Napoca, Romania

**Keywords:** surgical robot, virtual reality, control, simulator, single-incision laparoscopic surgery

## Abstract

In recent years, there has been an expansion in the development of simulators that use virtual reality (VR) as a learning tool. In surgery where robots are used, VR serves as a revolutionary technology to help medical doctors train in using these robotic systems and accumulate knowledge without risk. This article presents a study in which VR is used to create a simulator designed for robotically assisted single-uniport surgery. The control of the surgical robotic system is achieved using voice commands for laparoscopic camera positioning and via a user interface developed using the Visual Studio program that connects a wristband equipped with sensors attached to the user’s hand for the manipulation of the active instruments. The software consists of the user interface and the VR application via the TCP/IP communication protocol. To study the evolution of the performance of this virtual system, 15 people were involved in the experimental evaluation of the VR simulator built for the robotic surgical system, having to complete a medically relevant task. The experimental data validated the initial solution, which will be further developed.

## 1. Introduction

Applications using surgical robots are increasingly used in hospitals, found in an approximately 5%, although they were unequally distributed. Currently, these applications have multiple uses, such as abdominal, thoracic, neurosurgical, brachytherapy, pelvic procedures, and so on [1,2]. These values are quite different from one country to another based on financial stability, with a large increase in the number of surgical robots in the private medical sector. The skills of doctors play a vital role in performing surgical tasks through improved medical outcomes, patient safety, sensitivity, and increased accuracy. Just as aviation and military simulators have become standard for personnel training, so too may they have become for the surgical profession. Surgical interventions are performed with the help of robotic systems to support the surgeon perform these interventions with higher dexterity and faster responses to intraoperative complications [3].

The first robots used in surgical applications targeted specific parts of the intervention dealing with bone perforation in orthopedic surgery. In the 1990s, several robotic devices were introduced for simple tasks, such as the manipulation of the laparoscopic camera. A cornerstone in robotic surgery was the development of the first two full robotic platforms, Zeus and DaVinci, which, controlled from a master console, enabled the surgeon to perform the entire surgery by manipulating a set of instruments handled by the slave robotic platform. Following medical and commercial success, the da Vinci robotic platform has slowly spread throughout the world, even becoming a gold standard in many minimally invasive procedures. It must be pointed out that from a medical point of view, the robotic platform is a highly dexterous and accurate advanced tool with zero autonomy, with the entire procedure being performed by the surgeon [4].

Medically, surgical procedures evolved, also aiming to provide maximum therapeutic efficiency while minimizing the damage of healthy tissues. Thus, from classical, open surgery, in the 1980s, an important step was taken towards minimally invasive procedures, followed by the newer techniques which aim to perform the surgery through the anatomic orifices of the body, i.e., natural-orifice transluminal endoscopic surgery (NOTES) or through a unique access port, i.e., single-incision laparoscopic surgery (SILS) [5].

SILS is a developing technique due to its limitations in terms of surgeon ergonomics, instrument crossing, and limited working volume, but most of these negative aspects can be eliminated using a robotic platform dedicated to this task.

Surgeons need to develop their skills by practicing on robotic training systems that emphasize complex surgical scenarios, evaluations, and challenges for these skills. Such surgical training systems are available in two variants, namely: The first training approach is a classic method which involves the use of actual physical robotic systems on human phantoms, cadavers and then real patients (under the supervision of an expert in robotic surgery). This approach has a high cost and limited accessibility because a physical robotic system is and a mentor are needed to guide the future surgeon, while for live surgeries a second console must be used. The second training approach is a relatively new technique that uses VR to create simulators for robotic surgical training as a method to improve the robotic assisted surgical skills; it is derived from the gaming industry and with the technological progress it is now able to encompass high detail environments, relevant for advanced training programs. Furthermore, by using VR simulators, modifiable levels of difficulty and challenges can be used, where surgeons can also learn to manage unpredictable events [6]. Thus, these simulators are able to properly train surgeons before entering the operating room and working with patients. A second, very important advantage of these simulators is the preplanning of complex cases, when the digital twin of the patient is loaded and studied before the actual surgery to establish the most efficient approach [7]. Besides this, these simulators offer possibilities to implement new learning methods in a revolutionary way, yielding optimization of the acquisition of skills and their evaluation, leading to an overall risk reduction, enhanced medical outcomes, and efficient handling of any intraoperative complications.

This article describes a study in which a virtual reality simulator is developed for a medical robotic system for single-incision laparoscopic surgery (SILS). The novel character of this research consists of the implementation of a new robotic system in a VR environment and the use of multiple control strategies to perform the surgical act. This enables validation of the robotic structure before the experimental model is built, enabling its optimization based on the reported experience by the testers. The use of combined control modalities is also assessed. The robotic system consists of a parallel robotic module designed to manipulate a platform which carries three independent robotic modules for the manipulation of the specific instruments used in SILS procedures. The manipulation of the slave robotic system is carried out from a master console that embeds different end-effectors: a wristband multi-sensor used to actuate the active instruments and a set of voice commands proven to be a valid interaction solution for the laparoscopic camera [8]. For the validation of the surgical task involving the manipulation of the surgical instruments into the body of a virtual patient, a user must reach specific (predefined) points. The procedure time is recorded to be used as an efficiency indicator of the training process. The article is structured as follows: after the introduction, the next section provides the literature review, followed by the Materials and Methods section in Section 3. Section 4 presents the results and discussion, followed by Section 5 in which a summary and conclusions are given.

## 2. Literature Review

In the current literature, multiple applications can be found that use VR to create a highly immersive three-dimensional virtual environment that is used as a learning tool for systems found in the real environment. An example of such an application is found in a study that focused on developing/improving skills in robotic surgery using VR to train medical doctors in multidisciplinary surgery [9]. The study used the DaVinci simulator and included 24 exercises. The study was conducted over a period of 12 months, with the learning platform providing automated performance metrics and tracking learner progress. To complete the curriculum, a pre-test and post-test were required for the 21 students who participated in the study. After completing the curriculum, trainees reported improvements in their ability to operate the robotic system, and post-test scores were significantly higher than pre-test scores. Iop et. al. presented a systematic review of research related to VR in neurosurgery, with an emphasis on education. Five databases including thirty-one studies were investigated in this study after a thorough review process. This study focused on performance and user experience, showing that this technology has the potential to improve neurosurgical education using a wide range of both objective information and subjective metrics [10]. Korayem et al. discussed the possibility of using the Leap Motion Controller to directly control surgical robot arms and laparoscopic scissors during surgical procedures [11]. In [12], the authors performed a study where they trained 30 participants on how to configure a robotic arm in an environment that mimics the clinical setup. These participants were divided into three groups: one group was trained with paper-based instructions, one was trained with video-based instructions, and one was trained with VR-based instructions. By comparing the three methods, it emerged that the participants who used VR for learning gained a better understanding of the spatial awareness skills needed to achieve the desired robotic arm positioning. Mishra et al. presented a study that searched and reviewed articles showing numerous applications of VR/augmented reality (AR) in neurosurgery. These applications included their utility in the areas of diagnosis for complex vascular interventions, correction of spinal deformities, resident training, procedural practice, pain management, and rehabilitation of neurosurgical patients [13]. Covaciu et al. develop upper-limb rehabilitation simulators using VR [14,15]. Korayem et al. performed a study in which a vision-based contactless user interface named the Leap Motion Controller was presented. The device can track the speed, position, and orientation of the surgeon’s hand, being also able to detect the gestures and movements of each finger and then transfer data to the computer. Via this controller, a robotic arm that has a laparoscope attached is controlled [16]. Ehrampoosh et al. presented a new force-sensing instrument that facilitates teleoperated robotic manipulation and semi-automates the suturing task. The end-effector mechanism of the instrument has a rotating degree of freedom to generate the ideal needle-insertion trajectory and to pass the needle through its curvature [17]. In [18], a modular 3-degrees-of-freedom (3DoF) force sensor that easily integrates with an existing minimally invasive surgical instrument was presented. Abad et al. presented a haptic exoskeleton that attaches to the hand, consisting of five 4 × 4 miniaturized fingertip actuators (eighty actuators in total) to provide cutaneous feedback in laparoscopic surgeries, allowing the user to feel sensations in the form of vibrations produced by the actuators [19]. In [20], a hybrid parallel robot, called PARASURG 9M, was presented, consisting of a positioning and orientation module, with a kinematically constraint remote center of motion (RCM) and a dexterous active instrument with wide orientation angles for the distal head. A study was presented in [21] that demonstrated improvement in the performance of robotic surgery for beginners after training on a virtual reality simulator called RobotiX Mentor VR. The skills acquired during training are relevant to the use of the real robot in clinical practice because they have been transferred to a realistic model (avian tissue model). VR has also been used in the development of ankle rehabilitation simulators for people who suffered strokes. By attaching sensors to the limb, this simulator can interact with a real person. The data that are taken from the sensors are sent to an intelligent module to create new levels of exercises and control of the robotic rehabilitation structure in the virtual environment using machine learning [22]. VR is also used in the development of simulators in the control of a drone [23]. Luca et al. accomplished a study illustrating the potential of training using VR in spine surgery [24]. In [25], a meta-analysis was conducted in which it was shown that VR improves efficiency in trainee surgical practice. This study aimed to compare virtual reality with traditional training approaches, determining if it can complement or replace the training model. Performing research of the literature, 24 studies were highlighted that provided essential data. The results of the study suggested a positive effect that was observed during training with the VR simulator for controlling the laparoscope. Furthermore, this study highlighted that VR training emphasized crucial aspects for adequate surgical performance. Different VR systems offer multiple levels of difficulty, providing the student the opportunity to develop basic laparoscopic skills. Trochimczuk et al. carried out a study in which the concept of a novel telemanipulator for minimally invasive surgery was presented, and numerical analysis was carried out to validate the performance of the main system [26]. In [27,28], a review was made regarding the robots used in laparoscopic surgery. In [29], a study which mainly proposed to provide context for remote control of laparoscopic devices to improve the performance of minimally invasive surgical interventions was presented, so that all patients can have access to qualified surgeons even if they are in another region. First, with the help of the leap motion controller, the fine movements of the surgeons’ hands, the position and gesture of the fingers, and an awareness of the changes in the corresponding angles and coordinates, which were necessary at every moment, are acquired. To control the laparoscopic gripper, a 5-DOF robotic arm is built that is controlled using data from the Leap Motion sensor. Batty et al. presented the implementation of an environment estimation and force prediction methodology to mitigate system communication time delays for efficient implementation of haptic feedback in a minimally invasive robotic surgical system [30]. Mao et al. examined the current literature on the effectiveness of VR simulators regarding surgical skills in medical students, residents, and surgical staff. By examining the literature, it was concluded that trainees who used VR demonstrated an improvement in surgical skills, especially compared to those who used traditional non-VR methods [31]. In [32], a study was conducted that aimed to investigate the satisfaction of medical students regarding the training in robotic surgery offered at the Medical University of Varna, Bulgaria with the Da Vinci skills simulator. The results suggested that training in this field can be achieved even at the student level, using robotic surgery in realistic scenarios. Lamblin et al. conducted a study at the University of Lyon, France, in which they enrolled 26 junior specialist trainees to perform laparoscopic salpingectomy exercises on a VR simulator called LapSim. Junior trainees demonstrated that they improved their surgical skills following these exercises [33]. In [34], a study was conducted to evaluate the benefit of training with virtual reality simulation. The study was conducted at the ALEXEA (Alexandria Endoscopy Association) Center, in collaboration with the Department of Gynecology and Obstetrics, University Hospital, Campus Kiel Schleswig Holstein, Germany. The laparoscopy virtual reality simulator used was LapSim. The study concluded that virtual simulation could help teach basic skills in the early stages of training and provide a good simulation for procedural functioning for resident training. The virtual simulation demonstrated significant results in most parameters by a reduction in operating time, improvement in tissue handling, coordination of instruments, and reduction in the incidence of complications, resulting in an improvement in patient safety.

## 3. Materials and Methods

The innovative robotic system, PARA-SILS-ROB, was built based on the “master–slave” architecture, common for any surgical robotic system. This implies “zero” autonomy for the robot itself, all decisions and operations being performed by the surgeon. The master console includes all the necessary elements to enable full control of the surgical instruments, a task achieved through the slave robotic system. The VR simulation environment presented in this paper acts, from the user point of view, as a virtual master console where interfaces can be tested, user skills can be enhanced, and the slave robotic system, embedded as a digital twin, can be optimized in parallel before its actual development.

**Figure 1:**
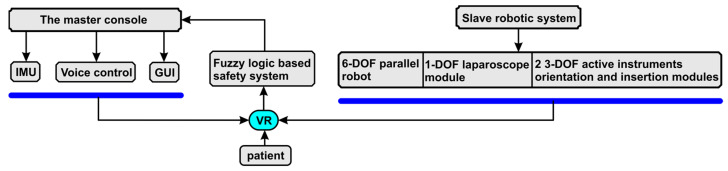
The SILS master–slave training system.

In Figure 1, the general architecture of the “master–slave” training system and its “virtual” counterparts are illustrated. The master console consists of the IMU sensor (having an accelerometer, gyroscope, and magnetometer); the voice control module, which is used to select the controlled module of the slave robotic system; other various elements of the VR environment (e.g., the viewing camera); and the graphical user interface (GUI) used as a backup solution of the IMU system and to provide additional parameters’ selection. The slave robotic system consists of three modules: the 6-DOF parallel robot, used to position the mobile platform (MP), on which the 1-DOF laparoscope module (for laparoscope insertion) and the two 3-DOF active instruments orientation and insertion modules, used to guide the active SILS instruments, are placed. According to medical protocol [35], the 6-DOF task is used to perform instruments’ modules registration, namely to control the mobile platform and position it at the SILS port, so that the remote center of motion (RCM) of the three mechanisms match the three corresponding trocar ports. After the registration, the MP is used for the orientation of the laparoscope, while the active instruments are controlled via the two 3-DOF modules. A virtual patient is placed within the VR environment, and a fuzzy logic system is developed to monitor their vital signs (heart rate, temperature, and oxygen level) and to generate specific alarms displayed on the operator GUI.

With focus on the VR environment, the next paragraph describes in detail the main components of the system from a hardware, software, and interconnectivity point of view.

### 3.1. The Slave Robotic System

**Figure 2:**
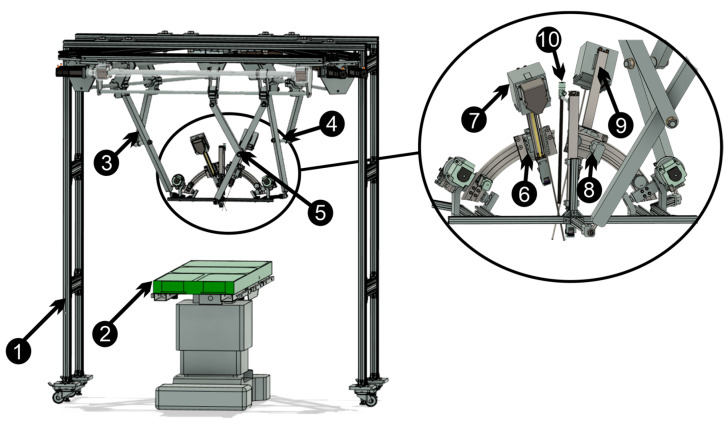
The parallel robotic structure and operating table: 1—framework; 2—operating table; 3—kinematic chain 1; 4—kinematic chain 2; 5—kinematic chain 3; 6—instrument orientation module 1; 7—active instrument 1; 8—instrument orientation module 2; 9—active instrument 2; 10—endoscopic camera.

The parallel robotic structure (Figure 2) has six degrees of freedom (DOF) with a modular construction consisting of three identical kinematic chains positioned along the sides of an equilateral triangle, connected to a mobile platform through three spherical joints. On the platform, three independent modules are embedded, each of them handing one instrument: the central module is a 1-DOF mechanism that performs the insertion/retraction of the endoscopic camera, while the lateral modules are two mechanisms with 3-DOF that have the role of achieving independent orientation of the mounted active instruments on the platform. The complete slave robotic structure contains the following main components:

- robot rigid frame;
- operating table;
- kinematic chain 1;
- kinematic chain 2;
- kinematic chain 3;
- instrument orientation module 1;
- active instrument 1;
- instrument orientation module 2;
- active instrument 2;
- endoscopic camera.

### 3.2. Singularity Analysis and Workspace of Parallel Robotic Structure

**Figure 3:**
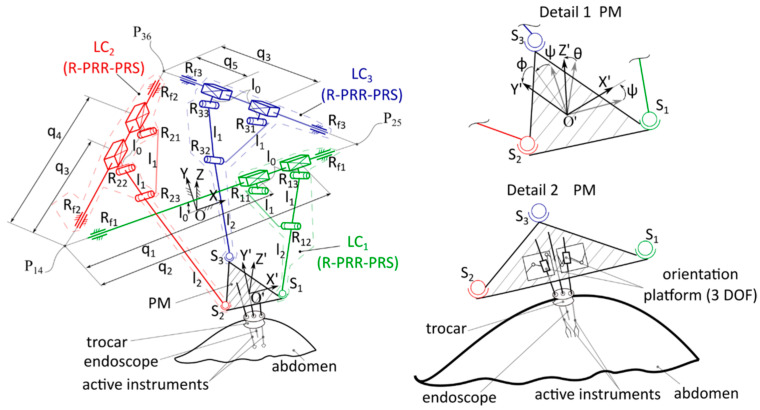
Kinematic diagram of the 6-DOF parallel robot type 3-R-PRR-PRS with triangular frame [41].

Robot singularity and workspace analyses are achieved to determine potential configurations where the mechanism could gain or lose degrees of freedom (becoming uncontrollable) and the operational working volume of the robotic structure in order to ensure safe conditions for the patient [36,37]. Singularity analysis of the 3-R-PRR-PRS parallel structure with a triangular frame and 6-DOF (Figure 3) is performed based on the kinematic model [38] of the structure, the CAD model being generated in the Siemens NX software, while the singularity positions of the structures are generated without assigning numerical values for the geometric parameters. Siemens NX software can also be used for finite element analysis (FEA) to determine the maximum deformation and the distribution of deformation [39], one of the critical factors in ensuring the safety operations of robotic systems working in the proximity of people [40]. Starting from the kinematic chain of the robot (Figure 3), the following are defined: LC_1_, LC_2_, and LC_3_ are the three kinematic chains (type R-PRR-PRS) that are actuated by the prismatic joints, namely q_1_, q_2_, q_3_, q_4_, and q_5_, q_6_. Each chain contains other three passive revolute joints: R_11_, R_12_, and R_13_ for LC_1_; R_21_, R_22_, and R_23_ for LC_2_; and R_31_, R_32_, and R_33_ for LC_3_. Each kinematic chain is connected through a passive spherical joint (S_1_, S_2_, S_3_) with the mobile platform, having the following geometric parameters: l_0_ represents the distance between the actuation axes, while l_1_ and l_2_ represent the mechanical links that compose the kinematic chains.

Following the solution of the kinematic model of the 3-R-PRR-PRS robot (with a triangular frame), the six expressions that represent the characteristic equations of the mechanism (Equation (1)) are determined, which are then be used to calculate the Jacobi matrices A and B [42].

(1) $$\begin{array}{l} f_{1}=q_{1}-q_{2}+2\cdot l_{1}\cdot \sqrt{1-\frac{d_{1}^{2}}{\left(l_{1}+l_{2}\right)}}=0\\ f_{2}:q_{2}-\sqrt{{\left(l_{1}+l_{2}\right)}^{2}-d_{1}^{2}}-d_{14\_S1}=0\\ f_{3}:q_{3}-q_{4}+2\cdot l_{1}\cdot \sqrt{1-\frac{d_{2}^{2}}{{\left(l_{1}+l_{2}\right)}^{2}}}=0\\ f_{4}:q_{4}-\sqrt{{\left(l_{1}+l_{2}\right)}^{2}-d_{2}^{2}}-d_{36\_S2}=0\\ f_{5}:q_{5}-q_{6}+2\cdot l_{1}\cdot \sqrt{1-\frac{d_{3}^{2}}{{\left(l_{1}+l_{2}\right)}^{2}}}=0\\ f_{6}:q_{6}-\sqrt{{\left(l_{1}+l_{2}\right)}^{2}-d_{3}^{2}}-d_{25\_S3}=0 \end{array}$$

Because the characteristic equations (Equation (1)) were defined to contain expressions in which the six active joints, qi, i = 1…6, are free terms of the first degree, the expression of matrix B is a very simple one, described in Equation (2). The determinant of matrix B is as follows: det(B) = 1, which leads to the statement that there are no singularities of type I.

(2) $$B=\left[\begin{array}{ccc} \begin{array}{cc} 1 & -1\\ 0 & 1 \end{array} & \begin{array}{cc} 0 & 0\\ 0 & 0 \end{array} & \begin{array}{cc} 0 & 0\\ 0 & 0 \end{array}\\ \begin{array}{cc} 0 & 0\\ 0 & 0 \end{array} & \begin{array}{cc} 1 & -1\\ 0 & 1 \end{array} & \begin{array}{cc} 0 & 0\\ 0 & 0 \end{array}\\ \begin{array}{cc} 0 & 0\\ 0 & 0 \end{array} & \begin{array}{cc} 0 & 0\\ 0 & 0 \end{array} & \begin{array}{cc} 1 & -1\\ 0 & 1 \end{array} \end{array}\right]$$

The determinant of matrix B is as follows: det(B) = 1, which leads to the statement that we have no singularities of type I. The determinant of matrix A has a complex form which, due to its large dimensions, is be illustrated in an explicit form. Applying several transformations and simplifications, this could be written as a product of 14 factors, which can be analyzed independently to determine type II singularity conditions:

(3) $$\det\left(A\right)=\prod_{i=1}^{14}F_{i}$$

The first factor has a numerical expression (Equation (4)), introducing no singularity conditions:

(4) $$F_{1}=108$$

Factors 4, 5, and 7 are terms that depend exclusively on geometric parameters, which can only theoretically become zero as all geometric parameters of the robot that represent lengths have positive values (Equation (7)):

(5) $$F_{6}=l^{3},F_{7}=l_{1}^{3},F_{9}=\frac{1}{{\left(l_{1}+l_{2}\right)}^{6}}$$

Further analysis of the other factors reveals that some have very complex expressions and, as such, they are analyzed. Therefore, factor 2 in its initial form has 1698 terms and degree 21, which is then transformed through combinatorial mathematical operations and brought to a more compact form, consisting of three terms, two of which are simple expressions, while the third has 330 of terms and degree 11. The first two resulting terms are as follows:

(6) $$F_{2\_1}=-1,F_{2\_2}=\cos\left(\theta \right)$$

If the first term does not introduce singularities, the second term introduces a singularity for $0=\pm \frac{\pi }{2},$ but this singularity is outside the operational workspace of the robot, for which, under special conditions, $\theta \in \left[-\frac{\pi }{3},\frac{\pi }{3}\right],$ and during the procedure, after inserting the instruments into the patient’s body, $\theta \in \left[-\frac{\pi }{6},\frac{\pi }{6}\right].$

For the evaluation of the polynomial equation defined by the third factor, MATLAB script was developed to evaluate the value of the function in the robot’s workspace, looking for the following information:

- Zero equality;
- Very small values of the evaluation result, which could suggest proximity to a singularity zone;
- Changes in the sign of the equation from one value to another, which could illustrate crossing through zero.

The results of these evaluations are presented below, where two examples are highlighted. In one, the values of the independent coordinates of the characteristic point of the mobile platform are varied, and in the second, its rotation angles varied between $\left[-\frac{\pi }{6}÷\frac{\pi }{6}\right]:$

**Figure 4:**
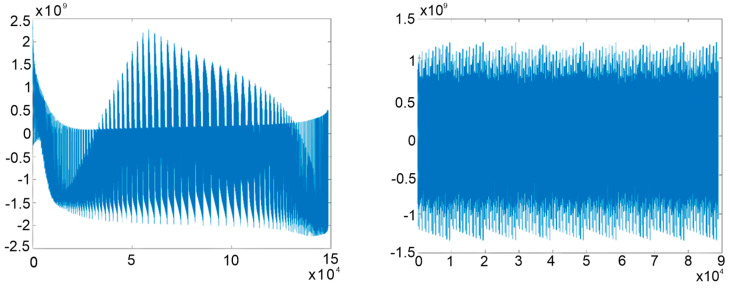
Graphical representation of the values of the term $F_{2\_3}$ for the two cases.

**Case 1.** Based on the graphical representation of the $F_{2\_3}$ values (computed with respect to the variation of the linear coordinates of the TCP), no points were identified in the robot workspace where the value of the factor was zero or close to zero. The range of variation was as follows:
  (7) $$\begin{array}{l} \min\left(F_{2\_3}\right)=-2.2353\cdot 10^{9}\\ \max\left(F_{2\_3}\right)=2.4313\cdot 10^{9}\\ \min\left(\left|F_{2\_3}\right|\right)=1.1629\cdot 10^{4} \end{array}$$
**Case 2.** Based on the graphical representation of the $F_{2\_3}$ values illustrated in Figure 4 (computed with respect to the variation of the linear coordinates of the TCP), no points were identified in the robot’s workspace where the value of the factor $F_{2\_3}$ was zero or close to zero. The range of variation was:
  (8) $$\begin{array}{l} \min\left(F_{2\_3}\right)=-1.3435\cdot 10^{10}\\ \max\left(F_{2\_3}\right)=1.2066\cdot 10^{10}\\ \min\left(\left|F_{2\_3}\right|\right)=2.9878\cdot 10^{4} \end{array}$$

Sign changes occurred only when switching from one set of input data values to another, which were generated between the minimum and maximum values. Thus, it can be stated that this term does not introduce singularities into the robot’s workspace.

The third factor is presented below:

(9) $$\begin{array}{l} F_{3}=3\cdot l\cdot \sin\left(\psi \right)\cdot \sin\left(\theta \right)\cdot \sin\left(\varphi \right)-3\cdot l\cdot \cos\left(\varphi \right)\cdot \cos\left(\psi \right)+3\sqrt{3}\cdot l\cdot \cos\left(\varphi \right)\cdot \sin\left(\psi \right)\cdot \sin\left(\theta \right)+\\ 6\sqrt{3}\cdot l_{instr}\cdot \cos\left(\theta \right)\cdot \sin\left(\psi \right)+\sqrt{3}\cdot l\cdot \cos\left(\theta \right)\cdot \sin\left(\varphi \right)+3\sqrt{3}\cdot l\cdot \cos\left(\psi \right)\cdot \sin\left(\varphi \right)+9\cdot L_{pf}+\\ 3\cdot l\cdot \cos\left(\varphi \right)\cdot \cos\left(\theta \right)-6\sqrt{3}\cdot Y_{E}-6\cdot l_{instr}\cdot \sin\left(\theta \right)-6\cdot X_{E} \end{array}$$

In this case, all the calculated values are positive for the entire operational workspace of the robot, which indicates that this term does not introduce singularities. 

The fourth factor is presented below:

(10) $$\begin{array}{l} F_{4}=-3\cdot l\cdot \sin\left(\psi \right)\cdot \sin\left(\theta \right)\cdot \sin\left(\varphi \right)+3\cdot l\cdot \cos\left(\varphi \right)\cdot \cos\left(\psi \right)+3\sqrt{3}\cdot l\cdot \cos\left(\varphi \right)\cdot \sin\left(\psi \right)\cdot \sin\left(\theta \right)-\\ 6\sqrt{3}\cdot l_{instr}\cdot \cos\left(\theta \right)\cdot \sin\left(\psi \right)+\sqrt{3}\cdot l\cdot \cos\left(\theta \right)\cdot \sin\left(\varphi \right)+3\sqrt{3}\cdot l\cdot \cos\left(\psi \right)\cdot \sin\left(\varphi \right)+3\cdot L_{pf}-\\ 3\cdot l\cdot \cos\left(\varphi \right)\cdot \cos\left(\theta \right)+6\sqrt{3}\cdot Y_{E}-6\cdot l_{instr}\cdot \sin\left(\theta \right)-6\cdot X_{E} \end{array}$$

In a similar way, the calculated values are entirely positive for the entire operational workspace of the robot, which indicates that this term does not introduce singularities either.

The fifth factor is presented next, with similar behavior to the previous two.

(11) $$F_{5}=2l\cdot \sqrt{3}\cdot \cos\left(\theta \right)\cdot \sin\left(\varphi \right)+6\cdot l_{instr}\cdot \sin\left(\theta \right)+3\cdot L_{pf}+6\cdot X_{E}$$

The terms $F_{8}$ and $F_{10}$, similar in content (but too bulky to represent), have similar behavior with no negative values for the entire operational workspace of the robot.

(12) $$\begin{array}{c} T_{{}_{11}}^{*}=2l\cdot \sqrt{3}\left(\sin\left(\varphi \right)\sin\left(\theta \right)l_{instr}\cos\left(\theta \right)+\sin\left(\varphi \right)\left(\cos\left(\psi \right)Z_{E}-\sin\left(\psi \right)Y_{E}\right)\sin\left(\theta \right)\right)\\ \;\;\;\;\;\;\;\;\;\;\;\;\;+2l\cdot \sqrt{3}\left(\cos\left(\varphi \right)\left(\cos\left(\psi \right)Y_{E}+\sin\left(\psi \right)Z_{E}\right)\right)+\left(-\cos{\left(\varphi \right)}^{2}l^{2}+l^{2}-3l_{instr}{}^{2}\right)\cos{\left(\theta \right)}^{2}\\ \;\;\;\;\;\;\;\;\;\;\;\;\;\;\;-6l_{instr}\left(\cos\left(\psi \right)Z_{E}-\sin\left(\psi \right)Y_{E}\right)\cos\left(\theta \right)-l^{2}-3Y_{E}{}^{2}-3\left(Z_{E}+l_{1}+l_{2}\right)\left(Z_{E}-l_{1}-l_{2}\right) \end{array}$$

The term $T_{11}^{*}$ represents the irreducible expression in the term $T_{11}$ that was again analyzed with respect to the operational workspace of the robot without registering values close to zero or changes in sign, with all values being positive. 

In the analysis of the term after simplifications, the following expression of the form is obtained:

(13) $$Exp=\sqrt{{\left(T_{12}^{*}\right)}^{2}}$$

Expression (12) yields elimination of the square root and raising to the power of 2 because the equation resolves the set of real numbers. Analysis of the remaining term, $T_{12}^{*}$, shows that it only takes positive values for all points considered in the operational space of the robot, with similar behavior encountered in the case of the next factor of the determinate, $F_{13}$.

By careful analysis, the last factor, $F_{14}$ has the following form (14):

(14) $$F_{14}=\frac{1}{\sqrt{{\left(F_{5}\right)}^{2}}}$$

By solving this equation in the set of real numbers, it leads to the simplification of the two factors, which leads to their elimination from the final expression of the determinant of matrix A. 

Thus, it can be concluded that for the operational space of the robot, which takes into account the orientation angles for the platform around the X and Y axes in the domain $\left[-\frac{\pi }{6}÷\frac{\pi }{6}\right]$, we have no singularity points, therefore ensuring safety conditions for the patient.

**Figure 5:**
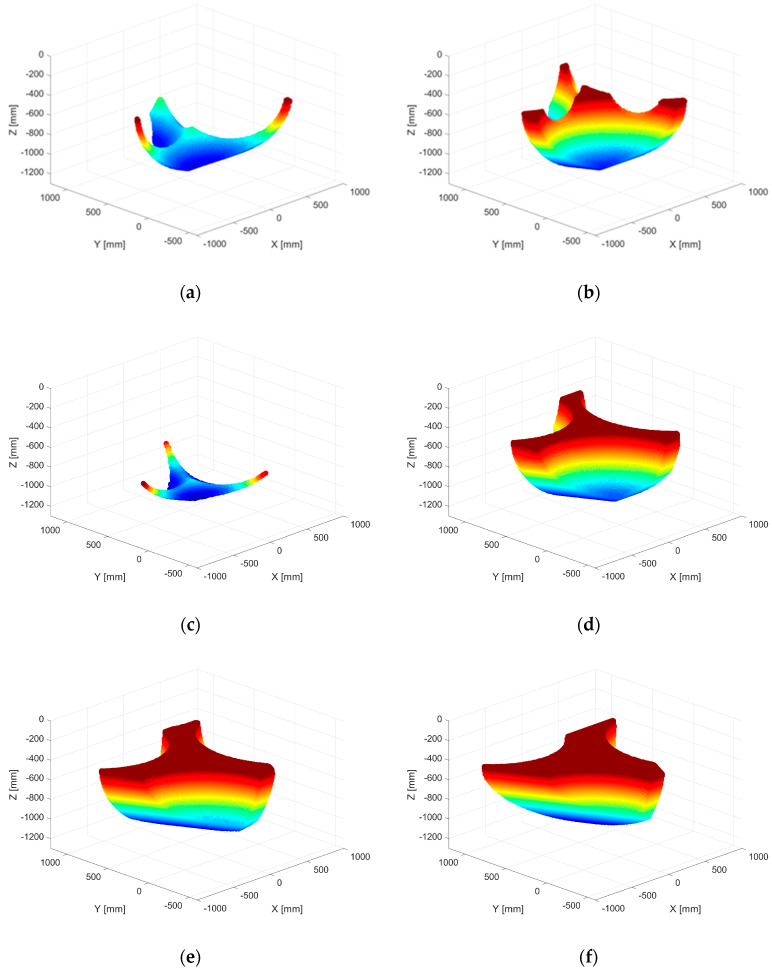
Workspace analysis of the 3-R-PRR-PRS parallel robot. (**a**) $\psi =0^{\circ },\;\theta =0^{\circ },\varphi =0^{\circ }$; (**b**) $\psi =0^{\circ },\;\theta =0^{\circ },\varphi =-30^{\circ }$; (**c**) $\psi =0^{\circ },\;\theta =0^{\circ },\varphi =30^{\circ }$; (**d**) $\psi =0^{\circ },\;\theta =0^{\circ },\varphi =-45^{\circ }$; (**e**) $\psi =0^{\circ },\;\theta =0^{\circ },\varphi =-60^{\circ }$; (**f**) $\psi =0^{\circ },\;\theta =0^{\circ },\varphi =-75^{\circ }$.

The workspace of the 3-R-PRR-PRS robot was generated using the inverse geometric model. The working space of the robot was calculated starting from the following geometric values of the main elements of the robot structure: l_PM_ = 760 mm; L_FP_ = 1260 mm; l_1_ = 375 mm; and l_2_ = 400 mm. Using a MATLAB script that embeds the inverse geometric model of the robot structure. Different configurations of the mobile platform attached to the robot structure were generated to study the following:

- The configuration with respect to the angle (φ), which was demonstrated in [42], to greatly influence the workspace size (Figure 5);
- The total workspace of the robot with respect to the required orientation angles for the endoscopic camera, namely:$\psi ,\theta \in \left[-45^{\circ }÷45^{\circ }\right]$.

In Figure 5, the workspace of the robot is evaluated for a vertical position of the laparoscopic camera (used for the initial steps of the procedure—when the camera is inserted into the patient), and it can be easily seen that the robot ensures the most efficient workspace when the angle (φ) is around the value of −60°.

**Figure 6:**
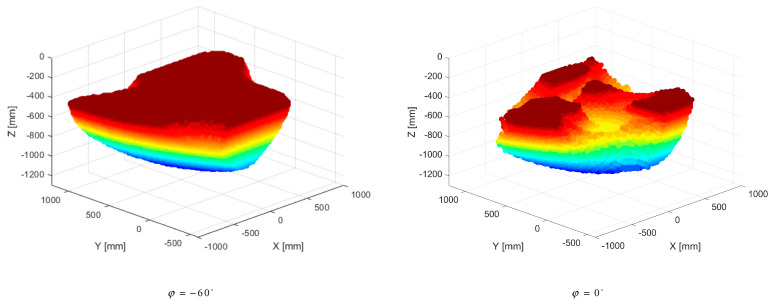
The total workspace of the 3-R-PRR-PRS parallel robot for φ= −60° versus φ = 0°.

Figure 6 illustrates the total workspace of the robot with respect to the SILS procedure where the coordinates of the endoscopic camera and the angles ψ and θ vary between minimum and maximum values, preserving the angle (φ) at the optimum value of −60°. Additionally, for validation, a second workspace is modelled with the angle (φ) at the “classical” value of 0°. The number of valid points for φ = −60° is 13.3 times higher than for φ = 0°, which points out the importance of using an efficient configuration for the robot platform (visible even from the density of points).

### 3.3. The Master Console

The master console consists of a set of hardware and software elements, which are used to control the slave robotic system. Through the different hardware interfaces, the user generates motion commands for the active instruments, which are processed by the software programs, generating the necessary movements at the level of the robot modules while providing real-time feedback through the endoscopic camera.

#### 3.3.1. The Multi-Modal Master Control Architecture

**Figure 7:**
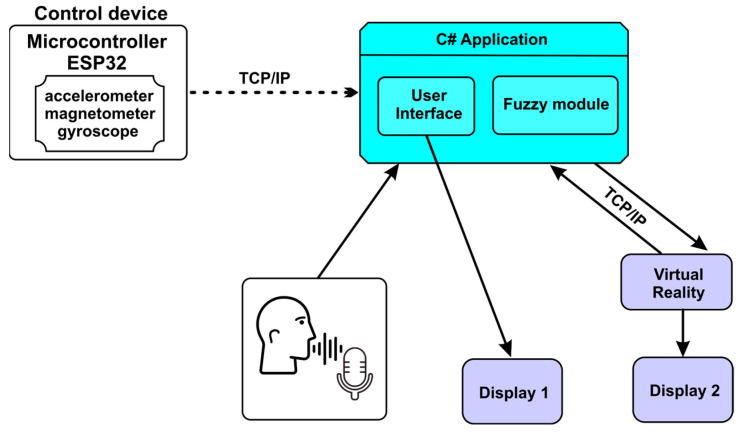
Interconnection of components.

The block diagram (Figure 7) shows the interconnection of the logical components in the control system. Control of the surgical robotic system can be performed through a device attached to the user’s forearm. This device contains a three-sensor module that includes a gyroscope, an accelerometer, and a magnetometer, and fusion of the three sensors is carried out for precise positioning. The control device is equipped with an ESP32 microcontroller used for data acquisition from the sensor module, and these data are sent to the user interface via the TCP/IP protocol to be processed. Voice recognition is used to change the control modules for the robotic system.

**Figure 8:**
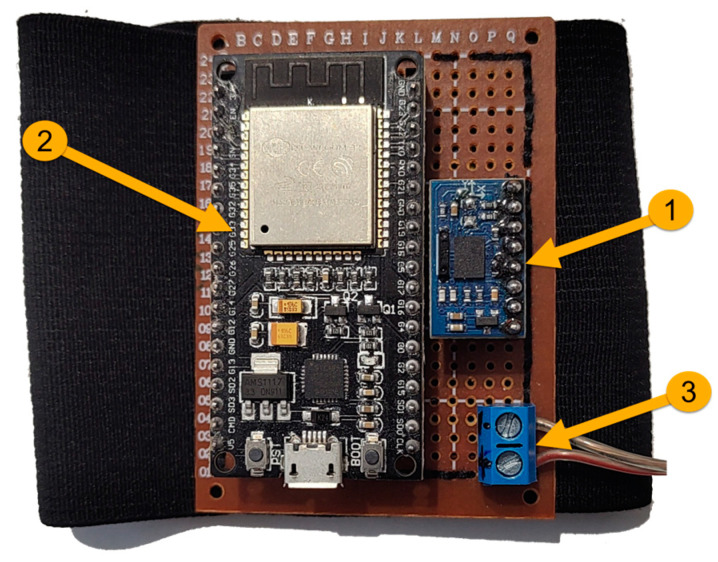
Control device: 1—Absolute orientation sensor, model IMU BNO055, 2—Microcontroller ESP32, 3—Power supply: 5V DC.

The control device (Figure 8) attached to the user’s forearm contains the following components:

- Absolute orientation sensor, which includes an accelerometer, magnetometer, and a gyroscope, model IMU BNO055 [43];
- Microcontroller ESP32 [44];
- Power supply: 5V DC.

#### 3.3.2. Software Application Development

**Figure 9:**
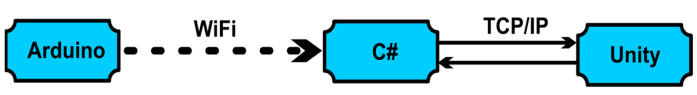
Software architecture.

The software applications are developed using two programming languages, namely C# (C Sharp) and Arduino. The Arduino programming language is used to implement the program that is stored on the ESP32 microcontroller for the control device (Figure 8) that attaches to the upper limb of the person controlling the surgical robotic system. The program stored on the ESP32 microcontroller receives data from the sensors embedded in the control device and encodes them in the format supported by the C# application, developed using the Visual Studio program. Data transmission from the microcontroller to the C# application occurs only when there is a change in the values provided by the sensors, being transmitted using the Wi-Fi network protocol. Both the user application and the VR application were written using the C# programming language, and the VR simulator was developed in the Unity development environment. Figure 9 shows the architecture of this application and how to establish communication between programs.

##### C# Software Analysis

**Figure 10:**
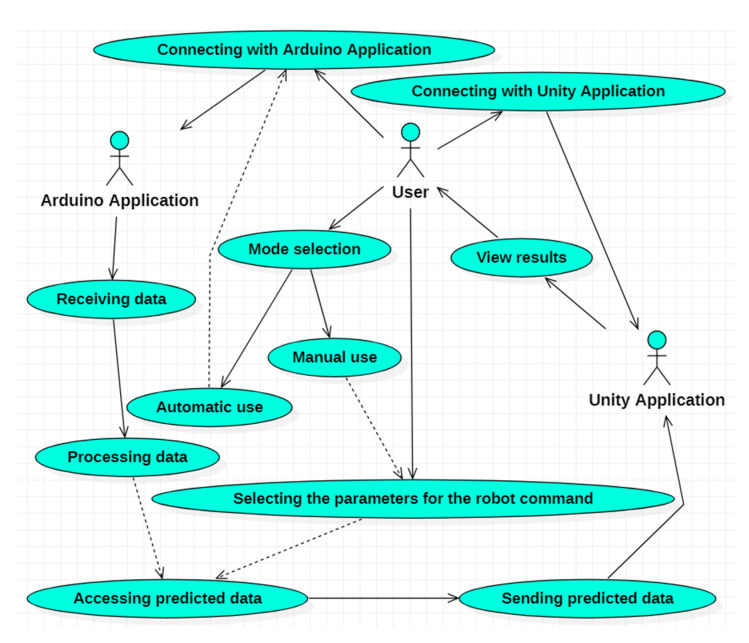
UML use case diagram.

A graphical depiction of the facilities offered by the software application can be made using UML diagrams [45]. In Figure 10, the use case diagram that indicates the functionalities of the software application implemented using C# programming language is presented [46]. This UML diagram is structured as follows:

- Eleven use cases that represent the functionalities of the C# software application.
- Three actors:
  ▪ The user or the external entity that interacts with the C# application.
  ▪ Script implemented in Arduino.
  ▪ Unity virtual reality application.
- Relations between the user and the use cases and relations between the use cases.

**Figure 11:**
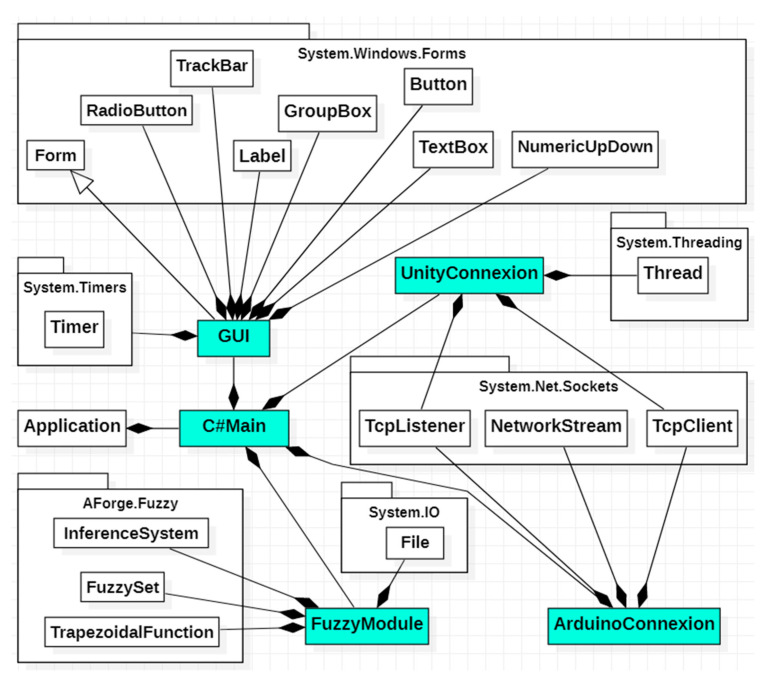
UML class diagram.

Starting from the functionalities presented in the use case diagram, five classes were designed and implemented, between which there are composition relations. The UML class diagram [47,48] represented in Figure 11 presents these five classes, the relation between them, and the used C# standard packages. These five classes are as follows:

- **GUI** class: enables the user to interact with the C# application. In order to realize the graphic interface for the user, eight classes from the System.Windows.Forms package are used.
- **ArduinoConnexion** class: establishes connection with the Arduino script and the transmission of the data acquired from the sensors.
- **UnityConnexion** class: establishes the connection with the virtual application developed in Unity.
- **FuzzyModule** class: implements a specific artificial intelligence algorithm [49], based on the fuzzy technique, to control the robot. For this purpose, three classes from the AForge.Fuzzy package are used: FuzzySet, TrapezoidalFunction, and InferenceSystem.
- **C#Main** class: constitutes the principal class of the C# application, consisting of four objects, i.e., one object of each previously presented class.

##### User Interface

The user interface was developed to control the surgical robotic system embedded in a virtual environment using VR technologies. The robotic system can be controlled via the user interface in two main modes:

- manually, by means of buttons and sliders on the user interface;
- automatically, using a control device equipped with sensors that attaches to the user’s upper limb, combined with voice control.

**Figure 12:**
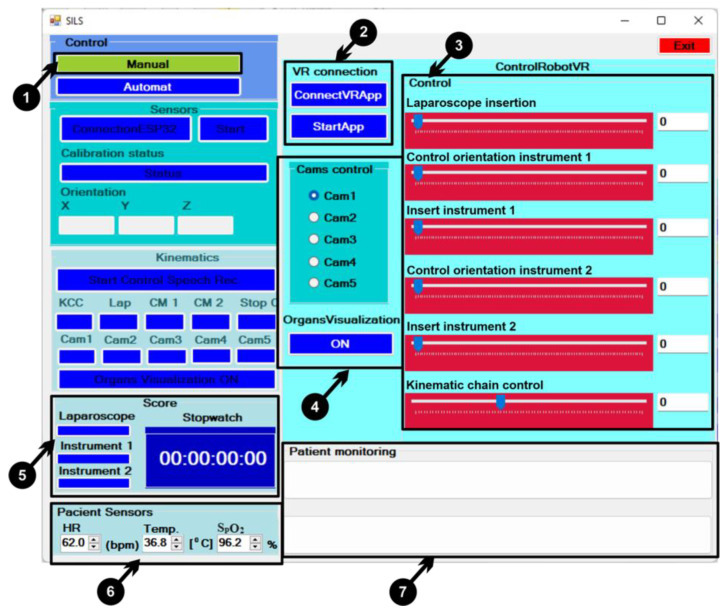
User interface: manual control.

The user interface was developed based on the features specified in the use case diagram, and the steps for using the interface are described in this section. For manual control, it is necessary to press the “Manual” button (Figure 12 (1)), which enables the manual control tools and disables the automatic controls for the user interface. The following commands are used for manual control:

▪ To establish the connection between the user interface and the virtual reality application through the TCP/IP protocol, the “ConnectVRApp” button must be pressed (Figure 12 (2));
▪ Data transmission between the user interface and the virtual reality application starts only after pressing the “StartApp” button (Figure 12 (2));
▪ Control of the robotic system is performed by means of sliders (Figure 12 (3)), as follows:
  ○ **Laparoscope insertion**: insert the laparoscope (Figure 2 (9)) into the virtual patient’s body;
  ○ **Control orientation instrument 1**: control the orientation of instrument 1 (Figure 2 (5));
  ○ **Insert instrument 1**: inserting instrument 1 into the body of the virtual patient (Figure 2 (6));
  ○ **Control orientation instrument 2**: control the orientation of instrument 2 (Figure 2 (7));
  ○ **Insert instrument 2**: inserting instrument 2 into the body of the virtual patient (Figure 2 (8));
  ○ **Kinematic chain control**: are controlled the kinematic chains of the robotic system structure (Figure 2 (3–5));
▪ For visualization in the virtual reality application from several angles, five viewing cameras can be set (Cam1...Cam5), and organ visualization in the virtual patient’s body is enabled by pressing the “ON” button (Figure 12 (4));
▪ By means of the sliders, the user can control the robotic system to insert the laparoscope and the two active instruments within a recorded time in defined points that are positioned on the kidneys, and when the three points are reached, the stopwatch stops and the elapsed time is recorded in a file (Figure 12 (5));
▪ Setting values for three virtual sensors (heart rate (HR), temperature, and oxygen(SpO_2_)) that are attached to the virtual patient (Figure 12 (6));
▪ Fields in which messages are displayed for monitor virtual sensor values and collisions when inserting instruments into the virtual patients.

**Figure 13:**
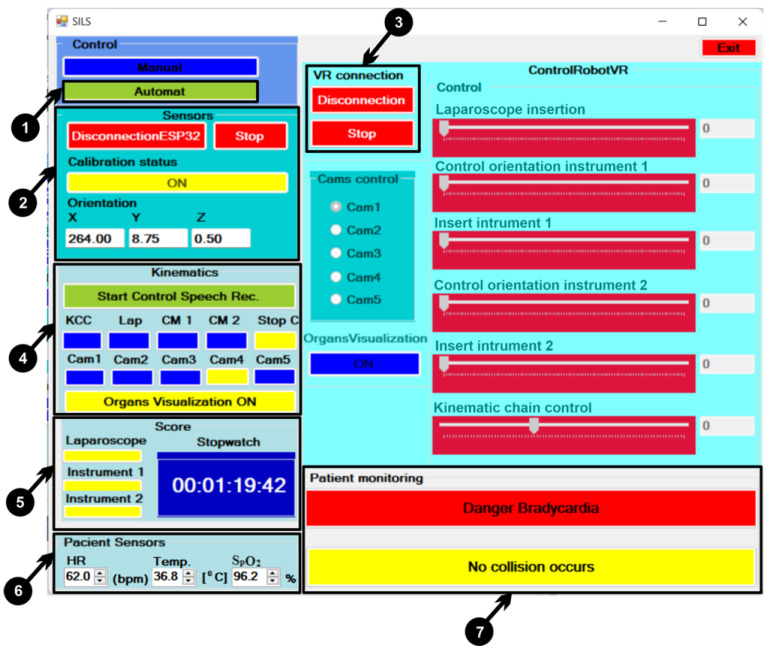
User interface: automatic control.

Automatic control is enabled in the user interface by pressing the “Automatic” button, which in turn disables the manual control tools. Switching the user interface to the automatic control mode can be carried out through the following steps:

▪ In order to create the connection between the microcontroller and the C# application (user interface) found on the computer via the Wi-Fi network protocol, the “ConnectionESP32” button must be pressed, and from that moment the “DisconnectionESP32” (Figure 13 (2)) status appears on the button on a red background;
▪ By pressing the “Start” button, the “Stop” status appears on the button (Figure 13 (2)), and from that moment the data from the microcontroller are transmitted to the C# application. For optimal functioning of the sensors, they are calibrated by moving the sensors of the device attached to the user’s upper limb on three axes; when the calibration is successfully executed in the “Calibration status” field, the status “ON” (Figure 13 (2)) appears on a yellow background. After the calibration is successfully accomplished, the sensors can be used to control the robotic system.
▪ In order to make a connection between the C# application and the virtual reality application via the TCP/IP protocol, the “ConnectVRApp” button must be pressed, and the virtual reality application starts, and from that moment the connection is created, and the status of the button reads “Disconnection” on a red background (Figure 13 (3)). In order to make data communication in both directions between the C# application and the virtual reality application, the “StartApp” button must be pressed, and from that moment the data communication starts, and the status of the button becomes “Stop” on a red background (Figure 13 (3)).
▪ In order to be able to use the control device with sensors that are attached to the user’s upper limb, the “Start Control Speech rec.” button must be pressed, and after pressing the button, its color changes to yellow-green (Figure 13 (4)). Pressing the button starts the stopwatch (Figure 13 (5)) and activates voice recognition commands that are combined with the sensors’ device commands to control the robotic system from within the virtual reality application. When activating the command via voice recognition, the color of the activated button changes from blue to yellow (Figure 13 (4)). The following describes these commands as follows:
  ○ **KCC** are controlled the kinematic chains of the robotic system structure (Figure 2 (3–5));
  ○ **Lap**: laparoscope control (Figure 2 (9));
  ○ **CM 1**: the module for controlling the rotation and insertion of the instrument 1 (Figure 2 (5,6));
  ○ **CM 2**: the module for controlling the rotation and insertion of the instrument 2 (Figure 2 (7,8));
  ○ **Stop C**: Stop control of the robotic system (Figure 13 (4));
  ○ **Cam 1**…**Cam5**: Five viewing cameras are used for different angles in the virtual reality application (Figure 13 (4));
  ○ **Organs Visualization ON**: command used to visualize the internal organs of the virtual human patient (Figure 13 (4));
▪ Upon touching each point positioned on the kidney by the laparoscope and the two active instruments, one LED lights up, and when both points have been successfully touched, the stopwatch stops (Figure 13 (5)), and the time is recorded in a file.
▪ Field for setting sensor (Figure 13 (6)) values (heart rate, temperature, and oxygen) that are attached to the virtual human patient;
▪ Field where messages are displayed regarding the condition of the virtual human patient from a medical point of view (Figure 13 (7)) by monitoring using three sensors (heart rate, temperature, and oxygen). In this field, messages alerting the user of the robotic system regarding the detection of collisions that may occur between the active instruments and the internal organs of the virtual human patient during the surgical procedure are also displayed.

##### Program for ESP32 Microcontroller

The embedded script for the ESP32 microcontroller was programmed and developed in an open-source programming environment called Arduino software (IDE) in the Arduino programming language. The following libraries were required in order to write the control script for the microcontroller:

- utility/imumaths.h: library for mathematical methods;
- Adafruit_Sensor.h, Adafruit_BNO055.h: library for BNO055 sensor use;
- Wi-Fi.h: library use for Wi-Fi network protocol;
- Wire.h: library for communication of ESP32 microcontroller with BNO055 IMU sensor.

After the C# application is connected to the Arduino script and while the connection is active, the data from the sensors are processed by the ESP32 script and encoded into a format supported by the C# application so that it can be sent later.

#### 3.3.3. Artificial Intelligence Based on Fuzzy Logic for Detecting and Avoiding Unforeseen Events

**Figure 14:**
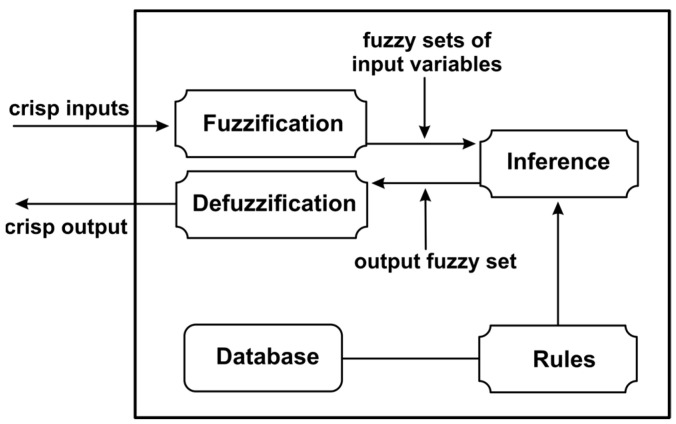
Block diagram of a fuzzy logic system.

Artificial intelligence agents based on fuzzy logic are used to create systems that detect and avoid unforeseen events. Fuzzy logic systems are an approach to variable processing that resembles human reasoning, with an approach that mimics human decision making. Fuzzy systems make an extension of classical sets by associating a function that returns a value between 1 and 0. Where it is difficult to implement a traditional control system, fuzzy logic can intervene. A block diagram of the fuzzy logic system can be seen in Figure 14. To activate rules, a fuzzification process is designed which transposes numerical expressions into fuzzy sets, which in turn associate fuzzy sets, corresponding to linguistic values. Rule manipulation is implemented by the inference motor which applies transformation of rule sets to fuzzy sets. To make a transformation from fuzzy sets to numerical values, the defuzzification process is used [50].

**Figure 15:**
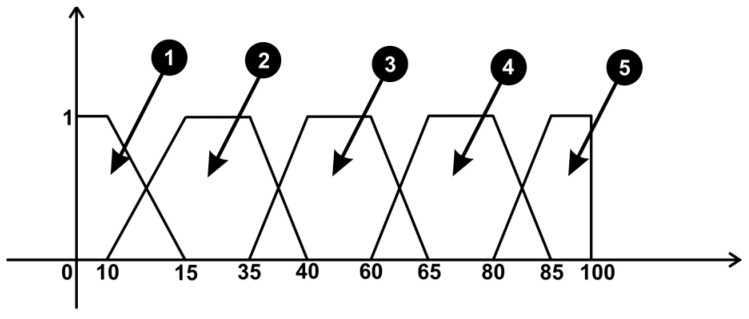
Fuzzy sets corresponding to events related to sensors.

To implement the fuzzy logic system, an open-source library called AForce.NET was used, implemented using the C# programming language. The scope of such a system in the entire surgical robotic application is to provide relevant information about the patient, considering the ongoing surgical operation. For this surgery application, two fuzzy logic systems were used as follows:

- For the first system, three virtual sensors (heart rate, body temperature, and blood oxygen level) were attached to the virtual patient and configured in such a way as to obtain distinct information about the patient’s biological signals by emitting visual signals of alarm (Figure 13 (7)) if these parameters are about to change in such a way that the patient’s life is endangered. Furthermore, a series of relationships between the parameters of these signals was created, starting from the premise that the change in the values of a signal can lead to the change in the values of another biological signal important for the safety of the patient, for example, the decrease in the level of oxygen in the blood can lead to tachycardia. The system can be integrated as suggestive behavior in the control of the robotic system, considering that any change in the patient’s medical condition can reconfigure the command the robot receives. The system architecture consists of three inputs: heart rate signal, temperature, and blood oxygen level. For prediction, the system has an output to display future events, as can be seen in Figure 15.

**Figure 16:**
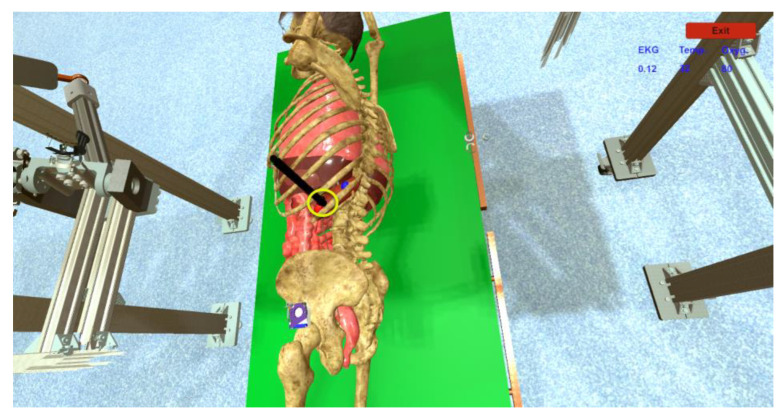
Laparoscope insertion.

The five output variables (Figure 15) are interpreted by the system as follows:

1. Between values 0 and 10, the output is “Danger Bradycardia”;
2. Between values 15 and 35, the output is “Bradycardia Alert!”;
3. Between values 40 and 60, the output is “Biological signals are within normal parameters”;
4. Between values 65 and 80, the output is “Tachycardia Alert!”;
5. Between values 85 and 100, the output is “Danger Tachycardia”.

- The second fuzzy system is used to detect collisions between the active tools and the organs of the virtual human patient (Figure 16). During the control of the robotic system when the three instruments are inserted into the body of the virtual human patient, these instruments may touch other organs than the desired ones and, as such, sets of rules are implemented that alert the user of the robotic system through messages (Figure 13 (7)) when an unwanted collision with another organ is happening or is about to happen.

**Figure 17:**
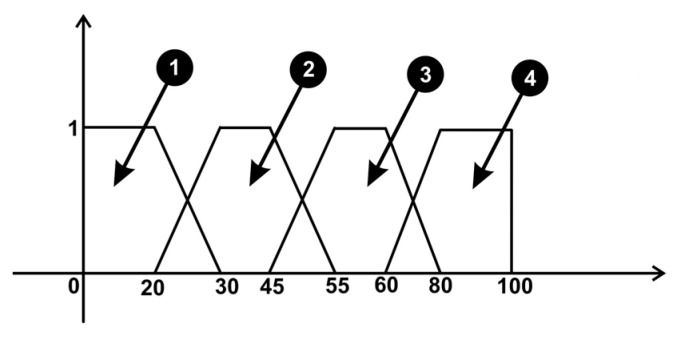
Fuzzy sets corresponding to events related to organs collision.

The architecture of the system consists of two inputs: collision ribs and collision internal organs. For prediction, the system contains an output for displaying events, as can be seen in Figure 17.

The four output variables (Figure 17) are interpreted by the system as follows:

1. Between values 0 and 20, the output is “No collision occurs”;
2. Between values 30 and 45, the output is “Danger: Rib collision!”;
3. Between values 55 and 60, the output is “Danger: Organ collision!”;
4. Between values 80 and 100, the output is “Danger: Rib and Organ collision!”.

#### 3.3.4. Virtual Reality Application

**Figure 18:**
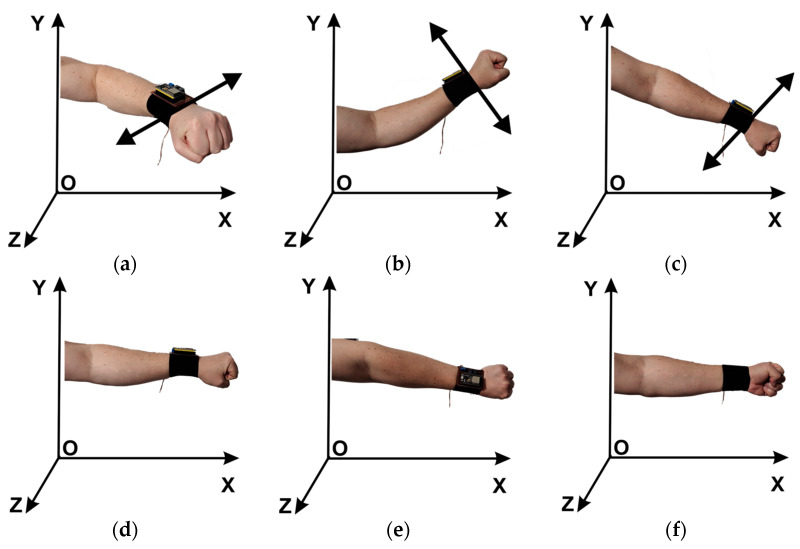
Rotation of the upper limb. (**a**) Around the Oy axis. (**b**–**f**) Around the Ox axis.

The parallel robotic system from virtual reality is designed to manipulate instruments using the single-incision laparoscopic surgery (SILS) procedure. This procedure represents a very good alternative for most minimally invasive procedures, offering a reduced hospitalization time [51], a shorter recovery time, and better aesthetic results compared to other procedures [52]. The virtual robotic system is controlled via the user interface by combining two types of controls. Before starting the VR application, the user attaches the control device to their wrist (Figure 2) and performs the calibration. To start the application from the user interface, the automatic control mode must be selected and the connection between the ESP32 microcontroller and user interface must be established. Pressing the “Start Control Speech Rec.” button activates the ability to control the robotic system through the following commands by voice recognition:

▪ **KCC**: the background becomes yellow, and the user can control the kinematic chain of the robotic structure (Figure 2, Figure 3 and Figure 4) by rotating the upper limb in the horizontal plane (parallel to the xOz plane) around the axis Oy (Figure 18a);
▪ **Lap**: the command is activated by voice recognition, the background becomes yellow (Figure 13 (4)), and the user can control the insertion and removal of the laparoscope (Figure 2 (9)) by a movement of rotating the upper limb in the vertical plane (parallel with the plane yOz) around the axis Ox of the upper limb (Figure 18b,c);
▪ **CM 1**: the background becomes yellow (Figure 13 (4)), and the user can control rotation module 1 (Figure 2 (5)) by a rotation movement of the upper limb in the vertical plane (parallel to the plane yOz) around the Ox axis (Figure 18e). After positioning rotation mode 2, the user can insert and remove active instrument 2 (Figure 2 (6)) through a movement of rotation of the upper limb in the vertical plane (parallel to the yOz plane) around the axis Ox of the upper limb (Figure 18b,c);
▪ **CM 2**: the background becomes yellow (Figure 13(4)), and the user can control rotation module 2 (Figure 2 (7)) through a movement of rotation of the upper limb in the vertical plane (parallel to the plane yOz) around the Ox axis (Figure 18,f). After positioning rotation mode 2, the user can insert and remove active instrument 2 (Figure 2 (8)) through a movement of rotation of the upper limb in the vertical plane (parallel to the yOz plane) around the axis Ox of the upper limb (Figure 18b,c);
▪ **Stop C**: the background becomes yellow (Figure 13 (4)), and the user stops controlling the robotic system;
▪ **Cam1**…**Cam5**: the background becomes yellow, and the user can change the viewing angle of the cameras;
▪ **Organs Visualization ON**: the background becomes yellow (Figure 13 (4)), and through this command the user can visualize the internal organs of the virtual human patient.

**Figure 19:**
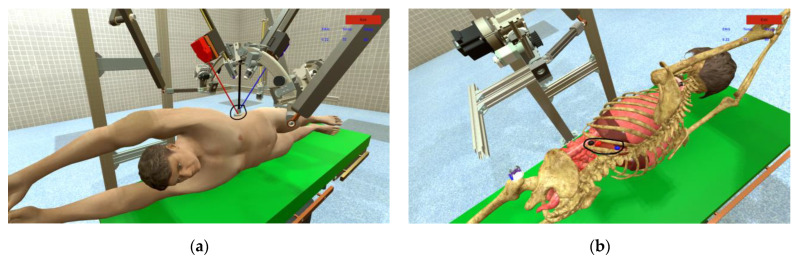
Operating the robotic surgical system. (**a**) Insertion of instruments through a trocar; (**b**) points to be reached by instruments.

When operating the robotic surgical system, the user must insert two active instruments and a laparoscope through a trocar that is positioned on the body of a virtual human patient (Figure 19a). Before the instruments can be manipulated, it is necessary to make a setting for three virtual sensors: heart rate, temperature, and oxygen from the “Patient Sensors” field (Figure 13 (6)). By changing the values of the sensors on the user interface in the “Patient monitoring” field, messages appear (Figure 13 (7)) in order to warn the user of the robotic system about possible medical problems. When manipulating the laparoscope and the two active instruments, accidental contact with internal organs can happen. These events are monitored by the fuzzy algorithm which reports any such event. For the user to be able to monitor the performance while operating the robotic system, three spheres are introduced that the user must touch with the laparoscope and the two active instruments (Figure 19b) while recording the time. When the targeted sphere is touched, the color of the background element changes to yellow for the three instruments (Figure 13 (5)). When the user successfully touches the spheres with the corresponding instruments, the timer is stopped.

## 4. Results and Discussion

### 4.1. Experimental Validation

#### 4.1.1. Participants

Fifteen healthy subjects (nine men and six women with a mean age of 31 years) participated in the experimental study after giving their informal written consent. To perform the experiment, all participants used their dominant upper limb and a headset with a microphone to control the robotic surgical system. Only two subjects knew the intention of the experiment, and the other subjects had no prior practice in controlling the surgical robotic system. Demographic details of the participants are shown in Table 1.

#### 4.1.2. Performance Evolution

In this study, the performance of each participant included in the experiment is analyzed. Before controlling the surgical robotic system, each participant attached a bracelet with sensors to their wrist (Figure 8).

**Figure 20:**
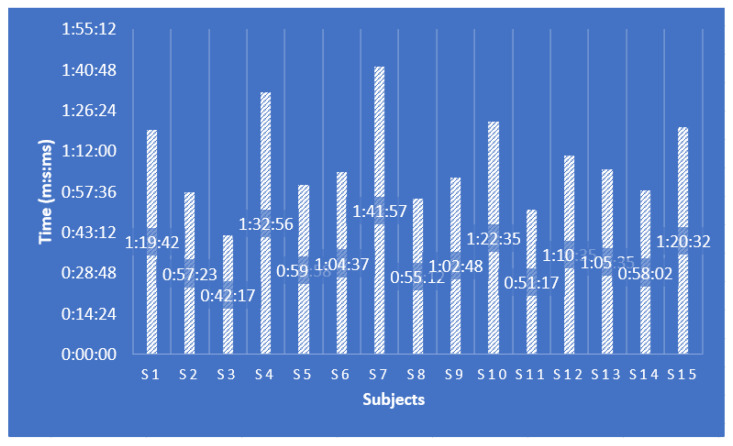
Performance results of users of the surgical robotic system.

The goal for the participants in this experiment was to operate the robotic system in such a way as to insert the two active instruments and the laparoscope through the trocar (Figure 19a) into the body of the virtual patient and reach three target areas marked by three spheres (each sphere is assigned to an instrument). When inserting the instruments, to be able to visualize the contact with the spheres, the “Organs Visualization ON” command is activated on the user interface (Figure 13 (4)), and from that moment the internal organs of the virtual human patient can be seen. Successful contact between the instrument and the target sphere is indicated on the user interface through an associated element by changing the background from blue to yellow (Figure 13 (5)). When the three spheres are touched by the three instruments, the timer stops, and the elapsed time is recorded in a file. Figure 20 presents the performance of each participant in the experiment.

## 5. Summary and Conclusions

This article presents a study for the development of a virtual reality simulator of a robotic system for single-incision laparoscopic surgery (SILS). The VR environment represents an efficient solution for the validation of different features of the robot and the assessment of different interfaces that can be used for slave robot control, as well as an efficient training environment for young surgeons. By also integrating a “digital twin” of the innovative robotic structure, dimensional optimization can be achieved by running different medically relevant scenarios.

The theoretical study on the singularities of the slave robotic platform demonstrated its feasibility for the surgical act in terms of safety in operation. Workspace analysis enabled the identification of the most efficient configuration of the robotic system to maximize its operational working volume.

From the master console, the user can interact with the virtual robotic system using either voice commands or a wristband attached to the forearm. While voice commands have been previously used in surgery, the use of a wristband is a new approach. The team aims to evaluate the stability of the solution, the user acceptance, and the learning curve to determine the feasibility of this approach. The VR platform also supports the loading of simulated patient data (heart rate, temperature, and oxygen level), which can reproduce different critical situations. In the first iteration of the VR simulator, a demonstrative exercise was implemented to test the user performance, to test the acceptance levels with respect to the wristband utilization, and to assess the motion capabilities of the robotic system.

Regarding future improvements, the assessment of accuracy during the use of the VR simulator by the users will be considered. A special set of simulated incidents during different stages of the procedure is also targeted for implementation to assess the reaction of surgeons in critical, unexpected conditions [53].

## 6. Patents

Pisla, D., Birlescu I., Vaida C., Tucan P., Gherman B., Plitea N.: Family of modular parallel robots with active translational joints for single-incision laparoscopic surgery, OSIM A00733/03.12.2021.

## Figures and Tables

**Table 1:** Demographic details specific to the participants included in the experiment.

Subject	Age	Gender
1	42	m
2	25	f
3	22	f
4	43	m
5	35	m
6	36	m
7	43	f
8	24	f
9	22	m
10	43	m
11	26	m
12	23	f
13	30	m
14	24	f
15	27	m

## Data Availability

Data sharing is not applicable to this article.

## References

[1] Benway B.M., Bhayani S.B., Rogers C.G., Dulabon L.M., Patel M.N., Lipkin M., Wang A.J., Stifelman M.D. (2009). Robot Assisted Partial Nephrectomy Versus Laparoscopic Partial Nephrectomy for Renal Tumors: A Multi-Institutional Analysis of Perioperative Outcomes. J. Urol..

[2] Tucan P., Vaida C., Horvath D., Caprariu A., Burz A., Gherman B., Iakab S., Pisla D. (2022). Design and Experimental Setup of a Robotic Medical Instrument for Brachytherapy in Non-Resectable Liver Tumors. Cancers.

[3] Plitea N., Hesselbach J., Vaida C., Raatz A., Pisla D., Budde C., Vlad L., Burisch A., Senner R. (2007). Innovative development of surgical parallel robots. Acta Electron. Mediamira Sci. Cluj Napoca.

[4] Pugin F., Bucher P., Morel P. (2011). History of robotic surgery: From AESOP^®^ and ZEUS^®^ to da Vinci^®^. J. Visc. Surg..

[5] Arkenbout E.A., Henselmans P.W.J., Jelínek F., Breedveld P. (2015). A state of the art review and categorization of multi-branched instruments for NOTES and SILS. Surg. Endosc..

[6] Vasudevan M.K., Isaac J.H.R., Sadanand V., Muniyandi M. (2020). Novel virtual reality based training system for fine motor skills: Towards developing a robotic surgery training system. Int. J. Med. Robot. Comput. Assist. Surg..

[7] Hagmann K., Hellings-Kuß A., Klodmann J., Richter R., Stulp F., Leidner D. (2021). A Digital Twin Approach for Contextual Assistance for Surgeons During Surgical Robotics Training. Front. Robot. AI.

[8] Vaida C., Pisla D., Plitea N., Gherman B., Gyurka B., Graur F., Vlad L., Pisla D., Ceccarelli M., Husty M., Corves B. (2010). Development of a Voice Controlled Surgical Robot. New Trends in Mechanism Science. Mechanisms and Machine Science.

[9] Gleason A., Servais E., Quadri S., Manganiello M., Cheah Y.L., Simon C., Preston E., Graham-Stephenson A., Wright V. (2022). Developing basic robotic skills using virtual reality simulation and automated assessment tools: A multidisciplinary robotic virtual reality-based curriculum using the Da Vinci Skills Simulator and tracking progress with the Intuitive Learning platform. J. Robot. Surg..

[10] Iop A., El-Hajj V.G., Gharios M., de Giorgio A., Monetti F.M., Edström E., Elmi-Terander A., Romero M. (2022). Extended Reality in Neurosurgical Education: A Systematic Review. Sensors.

[11] Korayem M., Vahidifar V. (2022). Detecting hand’s tremor using leap motion controller in guiding surgical robot arms and laparoscopic scissors. Measurement.

[12] Mehrfard A., Fotouhi J., Forster T., Taylor G., Fer D., Nagle D., Armand M., Navab N., Fuerst B. (2020). On the effectiveness of virtual reality-based training for surgical robot setup. Comput. Methods Biomech. Biomed. Eng. Imaging Vis..

[13] Mishra R., Narayanan M.D., Umana G.E., Montemurro N., Chaurasia B., Deora H. (2022). Virtual Reality in Neurosurgery: Beyond Neurosurgical Planning. Int. J. Environ. Res. Public Health.

[14] Covaciu F., Pisla A., Vaida C., Gherman B., Pisla D. Development of a Virtual Reality Simulator for a Lower Limb Rehabilitation Robot. Proceedings of the IEEE International Conference on Automation, Quality and Testing, Robotics (AQTR).

[15] Covaciu F., Gherman B., Pisla A., Carbone G., Pisla D. Rehabilitation System with Integrated Visual Stimulation. Proceedings of the European Conference on Mechanism Science.

[16] Korayem M., Madihi M., Vahidifar V. (2021). Controlling surgical robot arm using leap motion controller with Kalman filter. Measurement.

[17] Ehrampoosh A., Shirinzadeh B., Pinskier J., Smith J., Moshinsky R., Zhong Y. (2022). A Force-Feedback Methodology for Teleoperated Suturing Task in Robotic-Assisted Minimally Invasive Surgery. Sensors.

[18] Chua Z., Okamura A.M. (2023). A Modular 3-Degrees-of-Freedom Force Sensor for Robot-Assisted Minimally Invasive Surgery Research. Sensors.

[19] Abad A.C., Reid D., Ranasinghe A. (2022). A Novel Untethered Hand Wearable with Fine-Grained Cutaneous Haptic Feedback. Sensors.

[20] Pisla D., Gherman B., Plitea N., Gyurka B., Vaida C., Vlad L., Graur F., Radu C., Suciu M., Szilaghi A. (2011). PARASURG hybrid parallel robot for minimally invasive surgery. Chirurgia.

[21] Martin J.R., Stefanidis D., Dorin R.P., Goh A.C., Satava R.M., Levy J.S. (2020). Demonstrating the effectiveness of the fundamentals of robotic surgery (FRS) curriculum on the RobotiX Mentor Virtual Reality Simulation Platform. J. Robot. Surg..

[22] Covaciu F., Pisla A., Iordan A.-E. (2021). Development of a Virtual Reality Simulator for an Intelligent Robotic System Used in Ankle Rehabilitation. Sensors.

[23] Covaciu F., Iordan A.-E. (2022). Control of a Drone in Virtual Reality Using MEMS Sensor Technology and Machine Learning. Micromachines.

[24] Luca A., Giorgino R., Gesualdo L., Peretti G.M., Belkhou A., Banfi G., Grasso G. (2020). Innovative Educational Pathways in Spine Surgery: Advanced Virtual Reality–Based Training. World Neurosurg..

[25] Portelli M., Bianco S., Bezzina T., Abela J. (2020). Virtual reality training compared with apprenticeship training in laparoscopic surgery: A meta-analysis. R. Coll. Surg. Engl..

[26] Trochimczuk R., Łukaszewicz A., Mikołajczyk T., Aggogeri F., Borboni A. (2019). Finite element method stiffness analysis of a novel telemanipulator for minimally invasive surgery. Simulation.

[27] Kawashima K., Kanno T., Tadano K. (2019). Robots in laparoscopic surgery: Current and future status. BMC Biomed. Eng..

[28] Longmore S.K., Naik G., Gargiulo G.D. (2020). Laparoscopic Robotic Surgery: Current Perspective and Future Directions. Robotics.

[29] Korayem M., Vosoughi R., Vahidifar V. (2022). Design, manufacture, and control of a laparoscopic robot via Leap Motion sensors. Measurement.

[30] Batty T., Ehrampoosh A., Shirinzadeh B., Zhong Y., Smith J. (2022). A Transparent Teleoperated Robotic Surgical System with Predictive Haptic Feedback and Force Modelling. Sensors.

[31] Mao R.Q., Lan L., Kay J., Lohre R., Ayeni O.R., Goel D.P., de Sa D. (2021). Immersive Virtual Reality for Surgical Training: A Systematic Review. J. Surg. Res..

[32] Kalinov T., Georgiev T., Bliznakova K., Zlatarov A., Kolev N. (2023). Assessment of students’ satisfaction with virtual robotic surgery training. Heliyon.

[33] Lamblin G., Thiberville G., Druette L., Moret S., Couraud S., Martin X., Dubernard G., Chene G. (2020). Virtual reality simulation to enhance laparoscopic salpingectomy skills. J. Gynecol. Obstet. Hum. Reprod..

[34] Elessawy M., Mabrouk M., Heilmann T., Weigel M., Zidan M., Abu-Sheasha G., Farrokh A., Bauerschlag D., Maass N., Ibrahim M. (2021). Evaluation of Laparoscopy Virtual Reality Training on the Improvement of Trainees’ Surgical Skills. Medicina.

[35] Gherman B., Vaida C., Pisla D., Plitea N., Gyurka B., Lese D., Glogoveanu M. Singularities and Workspace Analysis for a Parallel Robot for Minimally Invasive Surgery. Proceedings of the IEEE International Conference on Automation, Quality and Testing, Robotics (AQTR).

[36] Wenger P., Chablat D. (2023). A Review of Cuspidal Serial and Parallel Manipulators. ASME J. Mech. Robotics..

[37] Pisla D., Plitea N., Videan A., Prodan B., Gherman B., Lese D. Kinematics and design of two variants of a reconfigurable parallel robot. Proceedings of the ASME/IFToMM International Conference on Reconfigurable Mechanisms and Robots.

[38] Franklin C.S., Dominguez E.G., Fryman J.D., Lewandowski M.L. (2020). Collaborative robotics: New era of human–robot cooperation in the workplace. J. Saf. Res..

[39] Tucan P., Vaida C., Plitea N., Pisla A., Carbone G., Pisla D. (2019). Risk-Based Assessment Engineering of a Parallel Robot Used in Post-Stroke Upper Limb Rehabilitation. Sustainability.

[40] Merlet J.-P. (2006). Parallel Robots.

[41] Pisla D., Gherman B., Tucan P., Birlescu I., Pusca A., Rus G., Pisla A., Vaida C. Application oriented modelling and simulation of an innovative parallel robot for single incision laparoscopic surgery. Proceedings of the ASME.

[42] Pisla D., Pusca A., Tucan P., Gherman B., Vaida C. (2022). Kinematics and workspace analysis of an innovative 6-dof parallel robot for SILS. Proc. Rom. Acad..

[43] https://ro.mouser.com/new/bosch/bosch-bno55-sensor/.

[44] https://www.espressif.com/sites/default/files/documentation/esp32_datasheet_en.pdf.

[45] Iordan A.E., Covaciu F. (2020). Improving Design of a Triangle Geometry Computer Application using a Creational Pattern. Acta Tech. Napoc. Ser.-Appl. Math. Mech. Eng..

[46] Iordan A.E. (2019). Optimal solution of the Guarini puzzle extension using tripartite graphs. IOP Conf. Ser. Mater. Sci. Eng..

[47] Levitin A. (2017). Algorithmic Puzzles: History, Taxonomies, and Applications in Human Problem Solving. J. Probl. Solving.

[48] Iordan A. Development of Interactive Software for Teaching Three-Dimensional Analytic Geometry. Proceedings of the 9th International Conference on Distance Learning and Web Engineering.

[49] Panoiu M., Panoiu C., Iordan A., Ghiormez L. (2014). Artificial neural networks in predicting current in electric arc furnaces. IOP Conf. Ser. Mater. Sci. Eng..

[50] Dumitrescu C., Ciotirnae P., Vizitiu C. (2021). Fuzzy Logic for Intelligent Control System Using Soft Computing Applications. Sensors.

[51] Dong B., Luo Z., Lu J., Yang Y., Song Y., Cao J., Li W. (2018). Single-incision laparoscopic versus conventional laparoscopic right colectomy: A systematic review and meta-analysis. Int. J. Surg..

[52] Pisla D., Carami D., Gherman B., Soleti G., Ulinici I., Vaida C. (2021). A novel control architecture for robotic-assisted single incision laparoscopic surgery. Rom. J. Tech. Sci. Appl. Mech..

[53] Aydın A., Ahmed K., Abe T., Raison N., Van Hemelrijck M., Garmo H., Ahmed H.U., Mukhtar F., Al-Jabir A., Brunckhorst O. (2021). Effect of Simulation-based Training on Surgical Proficiency and Patient Outcomes: A Randomised Controlled Clinical and Educational Trial. Eur. Urol..

